# Fast Quantitative LC-MS/MS Determination of Illicit Substances in Solid and Liquid Unknown Seized Samples

**DOI:** 10.1021/acs.analchem.1c03310

**Published:** 2021-11-29

**Authors:** Giuseppe M. Merone, Angela Tartaglia, Sandra Rossi, Francesco Santavenere, Elisa Bassotti, Cristian D’Ovidio, Martina Bonelli, Enrica Rosato, Ugo de Grazia, Marcello Locatelli, Fabio Savini

**Affiliations:** † Pharmatoxicology Laboratory, Hospital “Santo Spirito”, Via Fonte Romana 8, Pescara 65124, Italy; ‡ Department of Pharmacy, University of Chieti−Pescara “G. d’Annunzio”, Via dei Vestini 31, Chieti 66100, Italy; § R&D Department, Eureka Lab Division, Via Enrico Fermi, 25, Chiaravalle 60033, Italy; ∥ Department of Medicine and Aging Sciences, Section of Legal Medicine, University of Chieti−Pescara “G. d’Annunzio”, Chieti 66100, Italy; ⊥ Laboratory of Neurological Biochemistry and Neuropharmacology, Fondazione IRCCS Istituto Neurologico Carlo Besta, Via Celoria 11, 20133 Milan, Italy

## Abstract

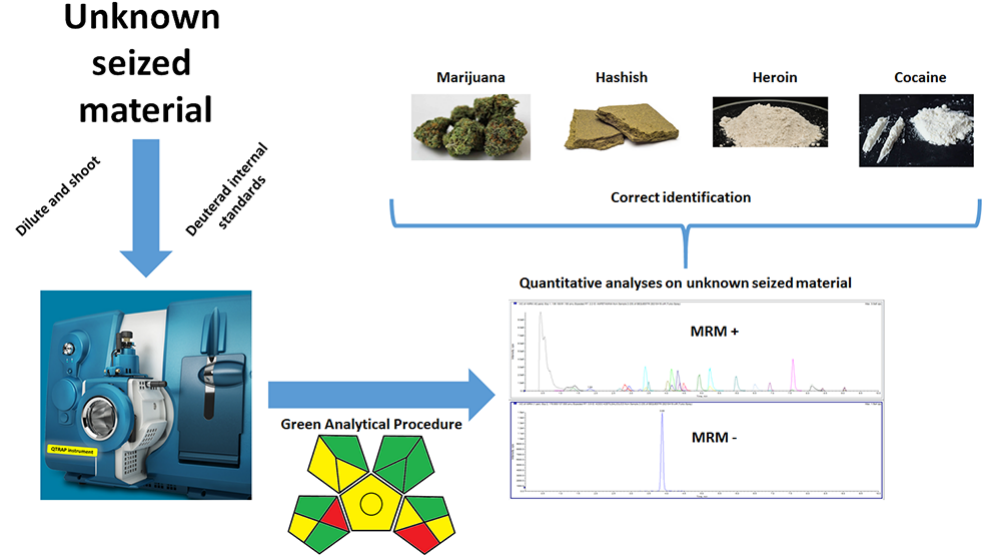

Narcotic and psychotropic substances are natural, synthetic, or semisynthetic compounds that are present in both solid and liquid illicit products. The alterations effects on the central nervous system related to their use can be psycholeptic, psychoanaleptic, or psychodiseptic and are able to generate tolerance, addiction, or dependence phenomena, creating social and public order problems. In this scenario, the analytical evaluations that aim to determine these analytes in seized nonbiological samples, and which assume the character of judicial evidence, must meet high analytical requirements of reliability, transparency, and procedures uniformity at a national level. For the first time in the literature, the herein validated method is able to provide the simultaneous quantitative determination of 37 of the most common narcotic substances as well as the most commonly used excipients/adulterants found in seized illicit material. Additionally, the validated method can process both solid and liquid samples maintaining the precision and trueness levels (intraday and interday) in accordance with the U.S. Food and Drug Administration and European Medicines Agency international guidelines (<14.31 and <13.41%, respectively). Furthermore, it provides a simple and fast procedure for sample preparation using the *dilute and shoot* approach, exploiting the sensitivity and selectivity of the LC-MS/MS instrument configuration used and the signal acquisition in multiple reaction monitoring (MRM) mode (both positive and negative polarization modes).

Narcotic and psychotropic substances are natural, synthetic, or semisynthetic compounds that show pharmacological activity and are able to alter the psychic and behavioral spheres to different degrees. The effects on the central nervous system (CNS) of these alterations can be psycholeptic, psychoanaleptic, or psychodiseptic. Often, these compounds could generate tolerance, addiction, or dependence phenomena. For these reasons, many substances, based on their different chemical structures, biological activities, and social effects, were included in national and international regulations as prohibited substances. In this context, these compounds are collected into specific tables which are constantly updated. As recently reviewed,^1,2^ the characterization of these seized substances can be extremely difficult, with long analysis and reporting times, especially if the laboratory does not have suitable protocols for the whole process and data traceability. In this framework, the analytical assessments, which aim to determine the narcotic and psychotropic substances in seized nonbiological samples and which assume the character of judicial evidence, must meet high analytical requirements of reliability, transparency, and procedures uniformity at national levels. During illicit substances analysis, a documentation in which the entire supply chain is traceable and compliant with current law regulations is always necessary. Alongside these requirements, it should also be added that high investigation levels must also be ensured through the instrumental techniques applications, procedures, and analytical methods that are robust and widely shared at the scientific community level in the toxicological and forensic fields. Generally, illicit seized samples often contain a wide range of other adulterating compounds that are added in order to increase the product bulk, facilitate administration, or even worse, mimic the pharmacological effects. These compounds can be both legal (caffeine, procaine, paracetamol, sugars, creatine, benzocaine)^3^ but also illegal (cocaine, 3,4-methylenedioxymethamphetamine or MDMA, amphetamines, mephedrone).^4−6^ Some examples of both solid and liquid unknown seized materials are shown in Figure 1. Currently, the works reported in the literature often consider only a limited quota of the possible substance combinations that can be found in seized samples^7−16^ by applying different instrumental configurations.

**Figure 1:**
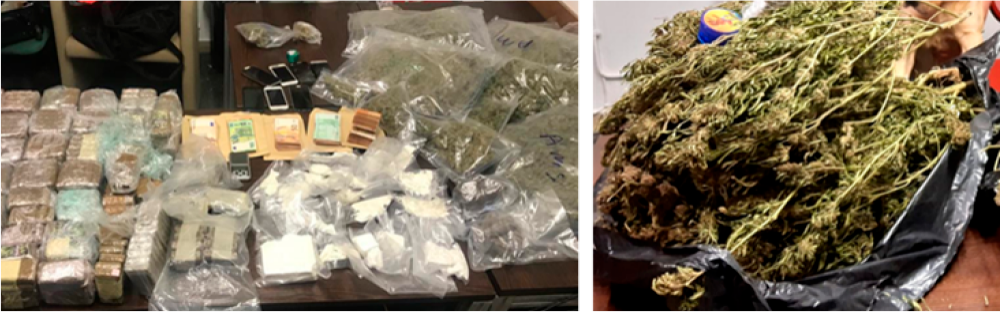
General appearance of some seized solid and liquid materials.

Our ongoing research aimed to improve the sampling and sample processing tool development^17−19^ by applying validated procedures useful for both clinical^20^ and legal^21^ purposes, coupled also with a more simple *dilute and shoot* concept;^22^ this method aims to provide a valid support in seized solid and liquid samples quantitative analysis. In particular, this method is able to simultaneously quantify 37 of the most common narcotic substances (cocaine, buprenorphine, amphetamine, methamphetamine, 3,4-methylenedioxymethamphetamine or MDMA, 3,4-methylenedioxyiamphetamine or MDA, 3,4-methylenedioxy-*N*-ethylphetamine or MDE, 3,4-methylenedioxy-*N*-methyl-α-ethylphenylethylamine or MBDB, ketamine, diacetylmorphine, ephedrine, pseudoephedrine, methadone, methorphan, 6-monoacetylmorphine or 6-MAM, delta-9-tetrahydrocannabinol or THC, cannabidiol or CBD, and morphine), as well as the most commonly used excipients/adulterants (acetylsalicylic acid, aminophenazone, benzocaine, caffeine, diltiazem, phenacetin, hydroxyzine, levamisole, lidocaine, naloxone, nicotine, noscapine, paracetamol, paroxetine, procaine, procainamide, trimethoprim, sulfametoxazole, tropacocaine).

## Experimental Section

### Materials and Instrumentation

The chemical standards used for the calibration curves, the QC samples, the HPLC mobile phases, and the solutions used in the sample extraction/dilution procedure were purchased from Eureka srl Lab Division (code LC20000). The complete list of the analytes (even with MRM transitions) is reported in Tables S.1 and S.2. The liquid chromatography-tandem mass spectrometry (LC-MS/MS) instrumentation is an ABSciex API 4500 QTrap interfaced with a Shimadzu Nexera X2 LC HPLC (SIL-30AC autosampler, LC-30AD pump and CTO-20AC column oven). The method was developed and validated at the Pharmatoxicology and Analytical Quality Laboratory (ACCREDIA n. 2274 ASLPE, accreditation n. 1822L, according to ISO/IEC 17025) of the “Santo Spirito Hospital”, Pescara, Italy. All configurations and instrumental parameters are detailed in Table S.3 and Figure S.1. Specifically, the mass spectrometer operating parameters (both for positive and negative ionization modes) are reported in Table S.4. The chromatographic column used, Hypersil Gold PFP (50 mm × 2.1 mm, 1.9 μm), was thermostated at 40 °C, while the analyses were carried out under gradient elution with binary phase M1 (H_2_O, 0.1% formic acid, 10 mM ammonium formate) and M2 (acetonitrile) with a flow rate of 400 μL/min according to the profile reported in Figure S.2. The analysis takes a total of 15 min including the system reconditioning step.

### Sample Preparation

The sampling phase on the seized materials was conducted following the guidelines on sampling of illicit drugs for the qualitative analysis of the European Network of Forensic Science Institutes (ENFSI),^23^ dividing the material into aliquots and weighing them accurately in order to provide a normalized quantitative analysis. Following the principle of less sample handling and taking advantage of the high LC-MS/MS configuration sensitivity and selectivity, sample preparation includes an extraction/dilution protocol for solid samples and a dilution protocol for liquid samples. In the case of seized solid samples: *(i)* weigh 20 mg of sample and add 5 mL of reagent A (acetonitrile), *(ii)* sonicate for 10 min and centrifuge for 10 min at 4000 rpm, and *(iii)* dilute 1:400 (*v:v*) with reagent B (H_2_O, 1% formic acid). At this point, for the quantitative determination of cocaine, proceed by preparing the sample in an autosampler vial by placing 10 μL of the sample solution in 990 μL of reagent D (H_2_O, 0.1% formic acid, 10 mM ammonium formate) and 20 μL of reagent C (methanol). The reagent C solution contains also the deuterated internal standards, specifically cocaine D3, 6-MAM D6, morphine D6, buprenorphine D4, methadone D9, and THC D3 (these internal standards were found to be adequate for all analytes in terms of parent ion affinity and retention times such as 6-MAM D6 for acetylsalicylic acid and morphine D6 for amphetamines). For all other substances, the volumetric ratios are 100 μL sample, 900 μL of reagent D, and 20 μL of reagent C. When it is necessary to analyze seizure liquid substances, the procedure involves only two steps: *(i)* add 10 μL of sample to 990 μL of reagent B and *(ii)* in autosampler vials 10 μL of the sample solution in 990 μL of reagent D and 20 μL of reagent C.

### Method Validation

The method was validated according to the U.S. Food and Drug Administration and European Medicines Agency international guidelines.^24,25^ and the following parameters were considered: linearity, lower limit of detection (LLOD), lower limit of quantification (LLOQ), precision and trueness (intraday and interday), and reagents and standards stabilities.

## Results and Discussion

### Extraction/Dilution and LC-MS/MS Procedures

**Figure 2:**
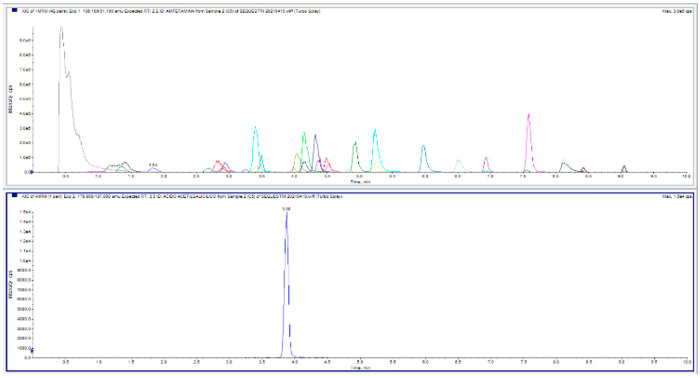
Chromatogram obtained under the optimized conditions for the standard solution at 100 ng/mL for all analytes and 120 ng/mL for paracetamol, caffeine, cocaine, methadone, phenacetin, and acetylsalicylic acid at concentration levels. The different colors represent the different 37 analytes MRM transitions. (Top) MRM in positive ionization mode. (Bottom) MRM in negative ionization mode.

First, the chromatographic separation reported in another study^17^ for the simultaneous analysis of more than 739 chemicals was tested. By applying the same chromatographic parameters, it was observed that the characteristics required, such as focusing the analytes in tight peaks to maximize the signal-to-noise ratio and consequently the sensitivity, avoiding the presence of matrix effects by resolving possible interferents through chromatography, and increasing the parameters for the correct identification considering also the reproducibility of the retention time, are maintained in the analyses of both liquid and solid seized samples. In this protocol, the first extraction of selected compounds was carried out with different solvents, such as methanol, isopropanol, hexane, dichloromethane, ethyl acetate, acetonitrile, and solutions formed by different percentages and combinations of the above-mentioned solvents. Different recovery factors were obtained, and the better performances were observed using acetonitrile that was selected for carrying out the first extraction. Then, this first extract was further diluted with organic solvents (methanol, acetonitrile) or with an aqueous solution in order to evaluate the better system to obtain the final sample ready for the analysis and to obtain better sensibility, signal-to-noise ratio, and ionization efficiency in the MS instrumentation and peaks symmetry during the chromatographic run. Dilution with an aqueous solution proved to be the most efficient. In addition, a percentage of formic acid was added because some molecules with acid pH were found to be more stable. The final dilution was focused on injection and chromatographic resolution in order to reach good peak shapes. It is very important that the injected solution is as similar as possible to the initial condition of the gradient in order to avoid peak tailings and non-Gaussian peaks shapes. The aqueous solution used for the final dilution was modified using only 0.1% formic acid and adding also 10 mM ammonium formate. The presence of 0.1% formic acid is very useful for peaks intensities. In this protocol, the parameters related to the analytes ionization and fragmentation are optimized. Electrospray ionization (ESI) temperatures were set from 250 to 550 °C with steps of 50 and 450 °C selected as the best one considering all molecules. Ion spray voltage is checked varying it from 500 to 5500 V, and +5400 and −4500 V are the best values (considering peaks intensities and noise) in positive and negative ionization modes, respectively. Regarding HPLC separation, it is very important to set a good mobile phase gradient, due to the presence of several isobar molecules with the same parent ions (MDE/MBDB, THC/CBD, MDA/phenacetin, ephedrine/pseudoephedrine, benzocaine), and some of them have also the same fragmentation (MDE/MBDB, THC/CBD, ephedrine/pseudoephedrine). For this reason, it is mandatory that their resolution is by HPLC gradient. The analysis was carried out using a gradient from 5% to 75% of organic solvent in 8 min to reach the separation between all these molecules. Acetonitrile and methanol are tried as organic solvents for mobile phase M2, and acetonitrile results to be the best one. In chromatographic separation development, in this case implemented to focus the analytes before their detection by MS/MS, it was also verified that no carry over (or memory effect) problems were present that could affect the batch analyzes through the use of an autosampler. No effects were found with the optimized mobile phase gradient, while maintaining the analysis within 15 min including the system reconditioning. The optimized LC-MS/MS parameters and the analytes chromatographic profiles, using polarity switching, are shown in Figure 2. More MRM transitions and instrumental parameters details are reported in Tables S.1–S.4 and Figures S.1 and S.2.

### Figure of Merits and Method Validation

The method validation procedure, obtained following international guidelines,^24,25^ saw the evaluation of analytical parameters such as linearity, lower limit of detection (LLOD), lower limit of quantification (LLOQ), precision and trueness (intra and interday), and reagents and standards stabilities. As reported in Table S.5, the method resulted linearly in the concentration range from 5 to 100 ng/mL with *r*^2^ values ≥ 0.9909, showing a lower limit of detection equal to 1.67 ng/mL. In the range, the performances of the methods were studied in terms of precision (CV%) and trueness (BIAS%) both intraday and interday (*n* = 6 for each sample and for each parameter). In the validated method, the calibration (once verified for the absence of matrix effects thanks to the *dilute and shoot* process) was carried out on standard solution samples. To validate the precision and trueness, solid and liquid samples (as they are and spiked) were analyzed with the procedure reported to have the background value subtracted from the instrumental response of the spiked sample. The result was evaluated in terms of precision and trueness for the quantity added and processed on the previously calculated calibration, obtaining the figures of merit reported in Table S.5. In particular, the precision was evaluated on three concentration levels and equal to LLOQ (5 ng/mL), *C*_m_ (50 ng/mL), and *C*_up_ (100 ng/mL). The performances in terms of trueness were evaluated at two concentration levels and equal to *C*_i_ (25 ng/mL) and *C*_h_ (75 ng/mL). Repeatability expresses the precision under the same operating conditions over a short interval of time and is also termed intra-assay precision. The intermediate precision expresses instead within-laboratory variations. As indicated by the precision and trueness values in Table S.5, the herein validated method has shown repeatability values that fulfill the international guidelines, as well as the intermediate precision. The recovery could be reported as trueness by the assay of a known added amount of analyte in the sample or as the difference between the mean and the accepted true value. In this work, the repeatability of the extraction process and the extraction yield indicated as intraday and interday trueness respects the limits for the methods validation. During the validation process, it was observed that all the reagents were stable up to 3 years at a temperature of 2–8 °C, while the chemical calibration standards (and QC) and reagent C containing the internal standards must be stored at −20 °C. No matrix effects were observed during the method development. This phenomenon can be ascribed to the fact that the *dilute and shoot* procedure developed and implemented provides for an overall dilution of the seized material by a factor of 1:10,000 (*w:v* in the case of seized solid materials and *v:v* in case of liquid). This high dilution factor, thanks to the instrumental sensitivity, completely reduces any effects on the analytes source ionization process, as well as minimize any effects related to peak asymmetries or fluctuations in the instrumental response.

### Real Sample Analyses

In order to demonstrate the method applicability, solid and liquid seized samples were analyzed. Some of more interesting analyzed samples are reported in Table 1, confirming the broad applicability of the reported method for the evaluation of unknown solid and liquid materials. Specifically, for the correct identification, we used the correspondences related to the known material, as reported in Figure S.3. In addition, in light of the procedure traceability due to the analyses in an accredited laboratory, the quantitative determination of up to 37 analytes, the easy execution and no analyte loss (related to the “dilute and shoot” process), the high selectivity and sensitivity of the instrumental configuration, together with an overall analysis time of 15 min, this method represents a very valid alternative to other procedures reported in the literature. A comparative evaluation is shown in Table S.6. In fact, compared to recent literature, this method provides an analysis time comparable with others (about 15–20 min), with highly sensitive and selective instrumentation (LC-MS/MS), with minimal sample handling (concept of *dilute and shoot*). However, the greatest advantage was certainly represented by the capacity to provide an accurate quantitative analysis (precise and trueness) through the use of internal standards that allow the normalization of the analyte signal. Another advantage was represented by the capacity to be applied both on solid and liquid samples without distinction while maintaining high sensitivity and reproducibility. The methods presented in the literature, in the case of quantitative analyses, consider a limited number of analytes compared to the present procedure (37 between narcotic substances and commonly used excipients/adulterants) and require laborious sample handling. In support of the importance of the LC-MS/MS configuration in the toxicological and forensic fields, it should also be emphasized that many recently developed devices are still based on the principles of mass spectrometry.^11^

**Table 1:** Quantitative Analyses on Real Liquid and Solid Seized Samples^a^

Presumed illicit substance	Founded illicit substance	Other substances	Active substance % (mg)
Cocaine (S)	Cocaine	–	99.0% (135,473)
MDMA (S)	MDMA	–	31,9% (129.4)
Marijuana (S)	THC	–	1.96% (52.6)
Hashish (S)	THC	CBD	14.1% (55.9)
		CBN	
Marijuana (S)	THC	–	2.84% (10.5)
Cocaine (S)	Cocaine	–	60.6% (155.0)
Cocaine (S)	Cocaine	–	53.0% (404.8)
Hashish (S)	THC	CBD	13.0% (311.8)
Heroin (S)	Diacetylmorphine, morphine, 6-monoacetylmorphine	Caffeine, noscapine, paracetamol	12.4% (240.0)
Heroin (S)	Diacetylmorphine, morphine, 6-monoacetylmorphine	Caffeine, noscapine, paracetamol	7.91% (21.8)
Hashish (S)	THC	CBD, CBN	14,9% (1442)
Marijuana (S)	THC	–	1.63 (867.0)
Cocaina (S)	Cocaine	–	88.0% (272.8)
Heroin (S)	Diacetylmorphine, morphine, 6-monoacetylmorphine	Caffeine, noscapine, paracetamol	3.82% (34.3)
Cocaine (S)	Cocaine	–	55.9% (126,044)
Marijuana (S)	THC	–	2.00% (18,692)
Hashish (S)	THC	CBD	12.0% (933.10)
		CBN	
Cocaine (S)	Cocaine	–	92.2% (165.1)
Heroin + Cocaine (Speedball) (S)	Diacetylmorphine, morphine, 6-monoacetylmorphine, cocaine	Caffeine, noscapine, paracetamol, tropacocaine	5.78% (12.8)
			25.8% (53.8)
Marijuana (S)	THC	–	4.56% (434.1)
Methadone (L)	–	Methadone	–
Cocaine (S)	Cocaine	–	48.8% (284.9)

a L, liquid seized sample; S, solid seized sample.

### Green Analytical Procedure Index (GAPI)

**Figure 3:**
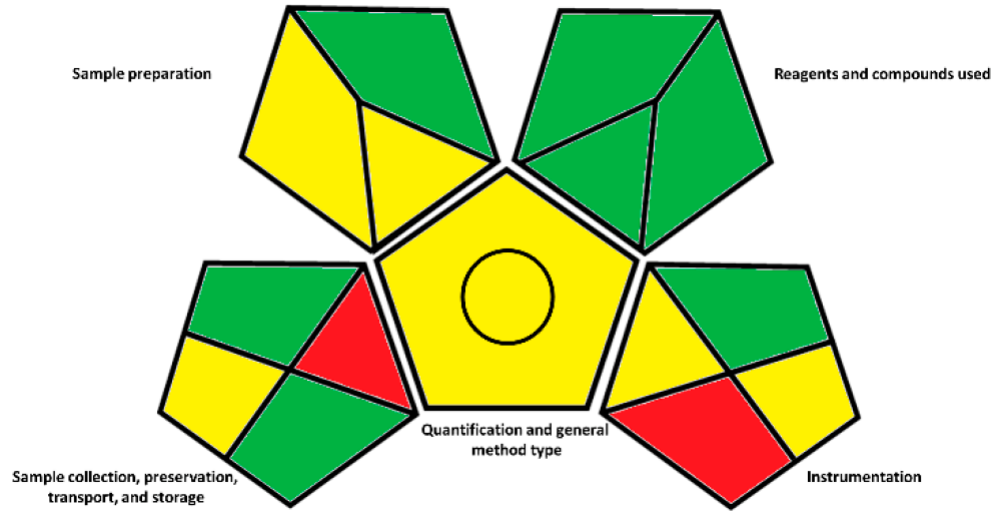
GAPI pictogram for the reported innovative device and procedure.

Lately, increasing importance has been given to the development of “green” methods. In this context, the objective is to reduce the anthropogenic activities impact on the environment, and in the case of analytical chemistry, it reflects the attempt to replace common organic solvents with nontoxic and nonpolluting ones. Other measures that refer to this trend can be found in the Principles of Green Chemistry.^26^ To date, to characterize the green profile of an analytical procedure, a reference could be made to the Green Analytical Procedure Index (or GAPI). For the herein reported method, as well as for others developed in our laboratory,^27^ we evaluated the eco-friendly profile by critically applying the principles that lead to the visualization of the GAPI pictogram in Figure 3. In particular, the details of the color assignment according to the method parameters are reported in Table S.7 and Figure S.4 and are based on the guidelines indicated by Płotka-Wasylka in 2018.^28^

## Conclusions

The major advantage of the herein reported procedure could be represented from the easy sample preparation process that followed the principle of *dilute and shoot*, avoiding the excessive sample manipulation. This simple procedure also included a step in which the deuterated internal standards were added and subsequently subject to LC-MS/MS analysis. This approach was made possible thanks to the high instrumental selectivity and sensitivity and through the signal acquisition in multiple reaction monitoring (MRM) mode. The herein validated methodology showed that LC-MS/MS represents the most suitable instrumentation to support law enforcement agencies (LEA) in terms of methods selectivity, sensitivity, and ruggedness in quantitative analyses, especially in the field solid and liquid seized samples analyses. However, it should be highlighted how other alternative methodologies (e.g., electrochemistry reported by Schram and collaborators^7^) can be of sure support for *on-site* analyses. Surely a possible limitation of the procedures in LC-MS/MS lies in the problems related to handling this instrumentation, which however are largely overcome by the advantages of its use for laboratory bench analysis. Moreover, it should be highlighted that the sample preparation process could be totally automated since, among all the preanalytical processes, the dilution steps are immediately transferable to automatic platforms. This step will certainly further increase the performances reported here, especially in regard to the reproducibility values.

## Supporting Information Available

Instrumental settings, detailed figure of merits for the validated procedure, chromatograms related to analyses of unknown seized materials performed on 19 April 2021, comparative table of this method with literature, and detailed information for the GAPI pictogram (PDF)
