# Supplementary material for: Fast Quantitative LC-MS/MS Determination of Illicit Substances in Solid and Liquid Unknown Seized Samples
Source: Anal Chem. 2021 Nov 29;93(49):16308–13. doi: 10.1021/acs.analchem.1c03310 (PMC8674870; doi:10.1021/acs.analchem.1c03310)

Fast quantitative LC-MS/MS determination of illicit substances in solid and liquid unknown seized samples

G.M. Merone [1,$], A. Tartaglia [2,$], S. Rossi [1], F. Santavenere [1], E. Bassotti [3], C. D'Ovidio [4], M. Bonelli [4], E. Rosato [2], U. de Grazia [5], M. Locatelli [2,*], F. Savini [1]

[1] *Pharmatoxicology Laboratory - Hospital "Santo Spirito", Via Fonte Romana 8, Pescara 65124, Italy;*

[2]*Department of Pharmacy, University of Chieti–Pescara "G. d'Annunzio", Via dei Vestini 31, Chieti 66100, Italy;*

[3] *R&D Department Eureka Lab Division, Via Enrico Fermi, 25, Chiaravalle 60033, Italy;*

[4]*Department of Medicine and Aging Sciences, Section of Legal Medicine, University of Chieti–Pescara "G. d'Annunzio", Chieti 66100, Italy;*

[5]*Fondazione IRCCS Istituto Neurologico Carlo Besta, Laboratory of Neurological Biochemistry and Neuropharmacology, Via Celoria 11, 20133 Milan, Italy.*

[$] *These authors contributed equally*

Corresponding author:

* Prof. Marcello Locatelli

Department of Pharmacy, University of Chieti–Pescara "G. d'Annunzio",
Via dei Vestini 31, Chieti 66100, Italy;
https://orcid.org/0000-0002-0840-825X; E-mail: marcello.locatelli@unich.it;
Phone: +3908713554590; Fax: +3908713554911.

Table of contents

Table S.1 Detailed MRM transitions monitored in this method

Table S.2 Internal standards MRM transitions

Table S.3 Chromatographic elution profile

Figure S.1 Gradient curve

Table S.4 Mass spectrometer parameters

Figure S.2 MRM in positive (upper) and negative (lower) ionization mode for the calibrators C5

Table S.5 Analytical parameters of the method for the 37 analytes

Figures S.3 Some chromatograms related to the analyses of unknown seized materials performed on 19th April 2021

Table S.6 Comparison with other methods in literature

Table S.7 General classification for GAPI index

Figure S.4 Correspondence to GAPI pictogram in the main text:

Table S.1 Detailed MRM transitions monitored in this method

Analyte	Internal Standard used as reference	Q1 (m/z)	Q3 (m/z) Quantifier	Q3 (m/z) Qualifier	Retention time (min)	Polarity
Cocaine	Cocaine D3	304.2	182.2	82.2	5.5	+
6-MAM	6-MAM D6	328.1	165.2	211.1	3.4	+
Amphetamine	Morphine D6	136.1	91.1	119.2	2.2	+
Methamphetamine	Morphine D6	150.2	91.1	119.2	3.2	+
MDA	Morphine D6	180.1	133.1	135.1	3.0	+
MDE	Morphine D6	208.2	163.1	135.1	4.5	+
MDMA	Morphine D6	194.1	163.1	105.1	3.7	+
MBDB	Morphine D6	208.2	135.2	177.1	4.6	+
Ketamine	Morphine D6	238.2	125.0	179.1	4.8	+
Methorphan	Cocaine D3	272.2	171.2	215.2	6.9	+
Buprenorphine	Buprenorphine D4	468.3	396.2	414.3	7.2	+
Methadone	Methadone D9	310.2	265.2	105.2	8.4	+
Morphine	Morphine D3	286.1	152.1	165.1	1.1	+
THC	THC D3	315.1	193.2	123.1	9.1	+
Aminophenazone	6-MAM D6	232.1	113.1	97.1	2.5	+
Caffeine	6-MAM D6	195.1	138.1	110.0	3.4	+
Diltiazem	Cocaine D3	415.2	178.2	150.1	7.2	+
Phenacetin	Cocaine D3	180.1	110.1	138.2	4.4	+
Hydroxyzine	Cocaine D3	375.2	201.1	166.1	7.8	+
Lidocaine	Cocaine D3	235.2	86.1	58.0	4.3	+
Naloxone	6-MAM D6	328.2	310.2	212.1	3.1	+
Noscapine	Cocaine D3	414.2	220.2	353.2	6.3	+
Paracetamol	Morphine D6	152.1	110.1	65.0	1.4	+
CBD	THC D3	315.3	193.1	123.1	8.4	+
Acetylsalicylic acid	6-MAM D6	179.0	137.0	93.0	10	-
Paroxetine	Methadone D9	330.2	69.9	192.2	7.8	+
Procaine	Cocaine D3	237.2	100.1	120.0	3.2	+
Procainamide	Cocaine D3	236.2	163.2	120.1	1.7	+
Sulfametoxazole	6-MAM D6	254.1	92.1	108.1	4.0	+

Table S.1 cont. Detailed MRM transitions monitored in this method

Analyte	Internal Standard used as reference	Q1 (m/z)	Q3 (m/z) Quantifier	Q3 (m/z) Qualifier	Retention time (min)	Polarity
Trimethoprim	6-MAM D6	291.1	230.2	261.2	3.6	+
Diacetylmorphine	Morphine D6	370.2	165.0	268.1	5.1	+
Levamisole	6-MAM D6	205.1	177.8	122.8	3.7	+
Tropacocaine	Cocaine D3	246.4	123.9	95.6	5.2	+
Benzocaine	Cocaine D3	166.1	138.0	77.0	4.6	+
Nicotine	Morphine D6	163.1	130.1	117.1	0.7	+
Ephedrine	6-MAM D6	166.1	148.4	91.1	2.1	+
Pseudoephedrine	6-MAM D6	166.1	148.0	91.1	2.7	+

Table S.2 Internal standards MRM transitions

Analyte	Q1 (m/z)	Q3 (m/z)	Retention time (min)	Polarity
Cocaine D3	307.2	185.2	5.5	+
THC D3	318.2	196.2	9.1	+
Methadone D9	319.2	268.2	8.4	+
Morphine D6	292.1	181.1	1.1	+
Buprenorphine D4	472.3	400.2	7.2	+
6MAM D6	334.1	211.1	3.4	+

Table S.3 Chromatographic elution profile

Time (min)	Flow rate (mL/min)	Mobile phase A	Mobile phase B
0		95	5
0.2		95	5
8		25	75
8.1	0.4	0	100
10		0	100
10.1		95	5
15		95	5

Mobile phase M1: H_2O, 0.1% formic acid, 10 mM ammonium formate;
Mobile phase M2: acetonitrile
Column: Hypersil Gold PFP (50 x 2.1 mm, 1.9 μm) thermostated at 40°C

Figure S.1 Gradient curve

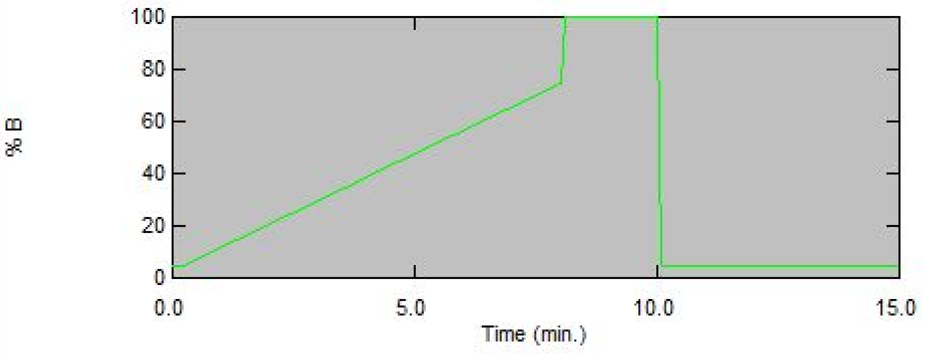

Table S.4 Mass spectrometer parameters

Ionization mode	Parameter	Value
POSITIVE	Curtain gas (CUR)	25
	Collision Gas (CAD)	Medium
	Ion Spray Voltage (IS)	5400
	Temperature (TEM)	450°C
	Ion Source Gas 1 (GS1)	55
	Ion Source Gas 2 (GS2)	55
NEGATIVE	Curtain gas (CUR)	25
	Collision Gas (CAD)	Medium
	Ion Spray Voltage (IS)	-4500
	Temperature (TEM)	450°C
	Ion Source Gas 1 (GS1)	55
	Ion Source Gas 2 (GS2)	55

Figure S.2 MRM in positive (upper) and negative (lower) ionization mode for the calibrators C5

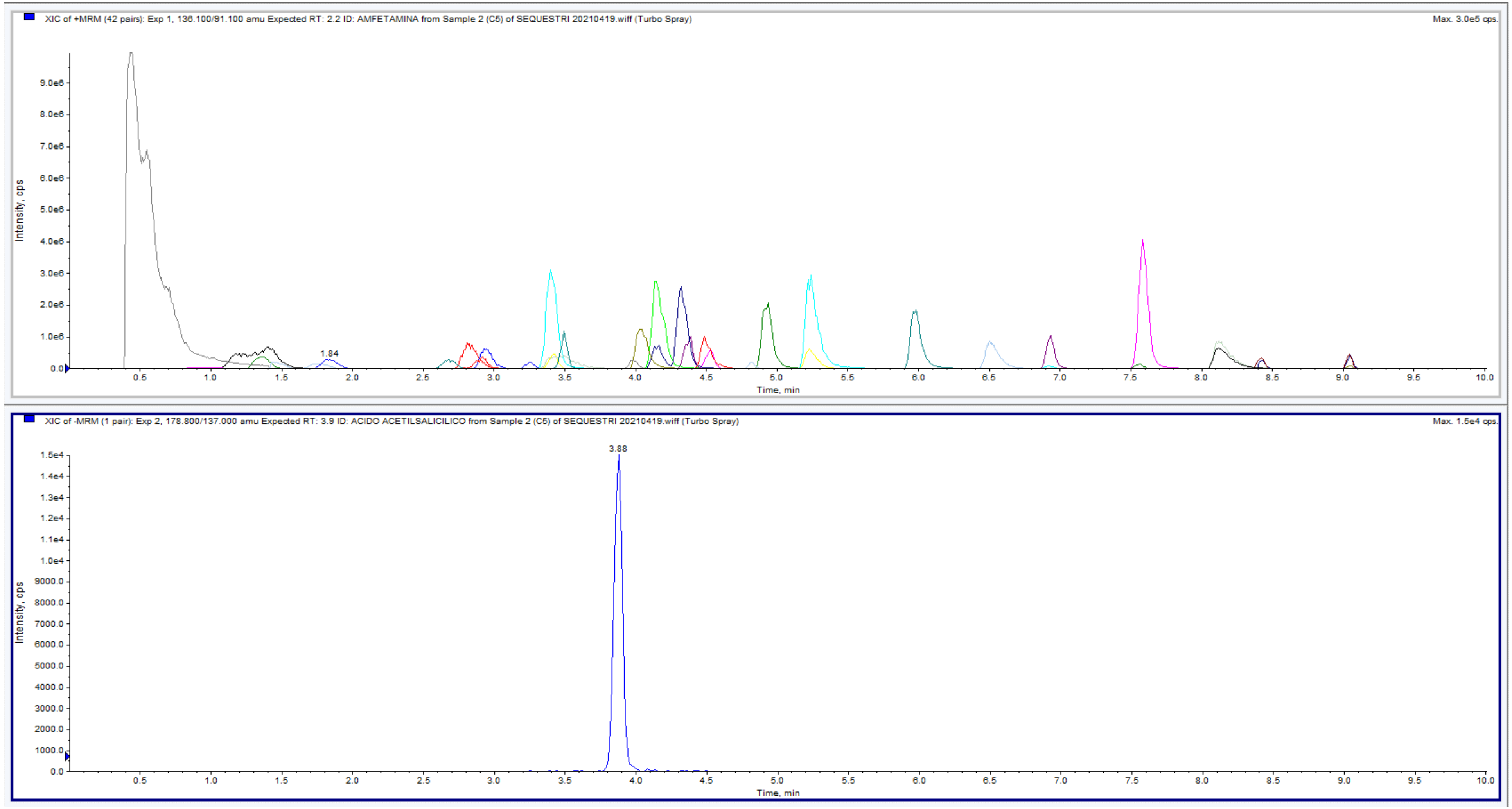

Table S.5 Analytical parameters of the method for the 37 analytes

Parameter	Range (ng/mL)	LLOD (ng/mL)	LLOQ (ng/mL)	Correlation Coeff. (r^2)[a]	Precision (CV%)						Trueness (BIAS%)			
					Intraday			Interday			Intraday		Interday	
					LLOQ	C_m	C_{up}	LLOQ	C_m	C_{up}	C_i	C_h	C_i	C_h
Cocaine				0.9945±0.0014	2.63	5.59	3.98	4.13	9.04	8.86	6.47	3.11	6.23	2.54
6-MAM				0.9942±0.0027	2.99	1.90	3.97	7.01	4.68	9.28	5.50	1.32	6.11	2.87
Amphetamine				0.9923±0.0012	3.55	4.87	2.08	9.91	9.88	8.60	7.46	5.16	8.88	6.01
Methamphetamine				0.9921±0.0021	6.57	7.84	3.66	8.67	9.12	7.57	10.8	1.77	11.0	1.90
MDA				0.9914±0.0017	1.31	2.88	5.36	7.75	6.30	8.95	6.58	2.71	7.59	3.58
MDE				0.9939±0.0014	6.74	5.84	5.36	10.7	6.78	8.72	6.76	2.70	10.9	3.36
MDMA				0.9945±0.0023	7.91	6.52	3.80	9.57	10.5	7.50	4.97	3.10	8.55	4.38
MBDB				0.9935±0.0019	2.80	6.97	3.33	9.15	8.08	6.70	6.94	2.82	9.73	3.77
Ketamine				0.9928±0.0018	4.25	7.28	4.79	9.41	8.32	8.33	6.69	2.56	9.73	3.96
Methorphan	5-100	1.67	5	0.9946±0.0030	3.43	5.10	3.77	7.10	5.65	8.65	6.91	1.90	7.67	4.39
Buprenorphine				0.9929±0.0011	4.54	4.01	2.11	5.78	8.03	6.65	5.91	0.35	7.08	3.12
Methadone				0.9966±0.0011	0.89	1.65	1.65	7.39	7.18	3.68	6.28	1.74	9.30	3.25
Morphine				0.9950±0.0019	3.91	4.94	0.85	7.49	7.95	8.12	4.88	2.16	8.26	2.79
THC				0.9952±0.0024	4.64	4.14	4.75	6.19	8.36	8.73	6.89	6.03	8.51	4.75
Aminophenazone				0.9929±0.0029	3.89	5.52	6.66	12.5	11.5	13.4	2.27	1.65	5.21	2.41
Caffeine				0.9919±0.0029	4.54	5.67	5.59	8.27	6.94	7.38	4.13	2.34	5.81	3.82
Diltiazem				0.9936±0.0021	1.50	5.05	3.49	5.52	8.69	7.89	4.40	2.16	9.15	2.55
Phenacetin				0.9943±0.0017	2.87	2.16	3.98	7.42	10.6	10.9	4.48	3.27	6.70	3.88
Hydroxyzine				0.9959±0.0028	2.08	1.59	1.53	6.57	5.98	3.69	13.4	7.10	11.2	9.88

Table S.5 cont. Analytical parameters of the method for the 37 analytes

Parameter	Range (ng/mL)	LLOD (ng/mL)	LLOQ (ng/mL)	Correlation Coeff. (r^2)[a]	Precision (CV%)						Trueness (BIAS%)			
					Intraday			Interday			Intraday		Interday	
					LLOQ	C_m	C_{up}	LLOQ	C_m	C_{up}	C_l	C_h	C_l	C_h
Lidocaine	5-100	1.67	5	0.9920±0.0031	5.95	7.68	3.51	8.66	9.91	7.69	5.80	7.91	9.68	8.00
Naloxone				0.9923±0.0023	4.69	5.23	6.08	12.8	9.89	11.4	2.83	3.50	6.83	4.41
Noscapine				0.9953±0.0033	4.01	7.14	5.27	5.46	8.60	9.73	1.31	1.75	4.51	2.70
Paracetamol				0.9909±0.0031	2.24	6.12	2.46	8.74	8.80	7.97	7.59	2.15	9.09	3.87
Ibuprofen				0.9924±0.0031	1.80	2.56	5.01	9.53	9.83	8.65	6.31	4.12	8.31	4.90
Acetylsalicylic acid				0.9978±0.0009	3.60	5.40	1.20	4.90	7.20	5.30	3.60	5.00	8.20	7.20
Paroxetine				0.9917±0.0068	3.73	3.79	4.97	5.71	7.39	7.55	7.08	3.19	8.82	6.08
Procaine				0.9922±0.0008	5.48	5.13	2.85	6.19	8.87	8.91	6.21	6.77	8.54	9.78
Procainamide				0.9929±0.0015	2.27	4.75	3.39	9.80	8.72	9.81	9.23	6.55	13.4	8.36
Sulfametoxazole				0.9939±0.0020	1.57	5.14	4.94	7.44	8.40	8.82	4.38	5.00	6.51	5.66
Trimetoprim				0.9904±0.0016	5.54	5.18	6.00	6.10	9.12	7.34	7.27	8.85	9.49	10.7
Diacetylmorphine				0.9920±0.0065	2.85	7.09	3.07	5.99	7.50	8.68	6.84	1.87	8.06	6.78
Levamisole				0.9921±0.0035	3.31	5.79	4.71	9.47	10.8	9.86	10.1	4.70	10.3	9.03
Tropococaine				0.9915±0.0055	4.16	5.94	2.65	4.52	7.49	9.34	5.65	2.29	7.35	4.19
Benzocaine				0.9950±0.0043	4.89	7.74	3.46	7.70	8.07	9.46	3.78	2.20	6.85	3.95
Nicotine				0.9945±0.0011	3.94	7.46	5.89	4.16	6.47	6.27	8.20	2.30	9.15	5.60
Ephedrine,				0.9911±0.0064	5.78	7.80	6.77	14.3	7.84	9.25	2.92	1.27	7.53	9.16
Pseudoephedrine				0.9911±0.0064	6.60	8.10	7.30	10.5	9.60	10.2	3.50	3.00	8.20	8.60

[a] Average of six determinations ± standard deviation; C_l = lower concentration 25 ng/mL; C_h = higher concentration 75 ng/mL; LLOQ = lower limit of quantification 5 ng/mL; C_m = medium concentration 50 ng/mL; C_{up} = upper concentration 100 ng/mL; LLOD = lower limit of detection.

Figures S.3 Some chromatograms related to the analyses of unknown seized materials performed on 19th April 2021

Transitions used for the identification of unknown seized material:

Cocaine	→	Cocaine + Cocaine D3
Marijuana/hashish	→	CBD, THC + THC D3
Heroin	→	6MAM + 6MAM D6, caffeine, morphine, noscapine, paracetamol

Identified material: COCAINE

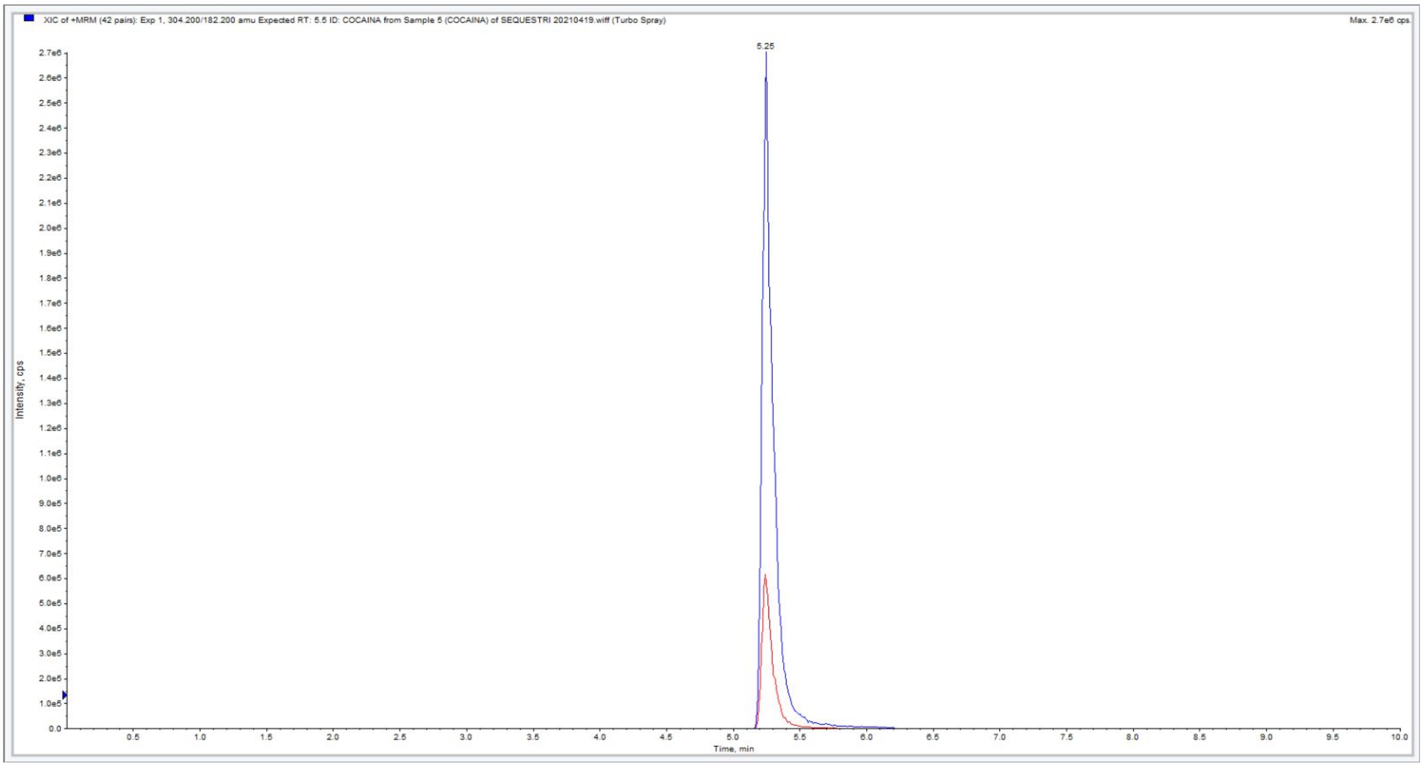

Identified material: HASHISH

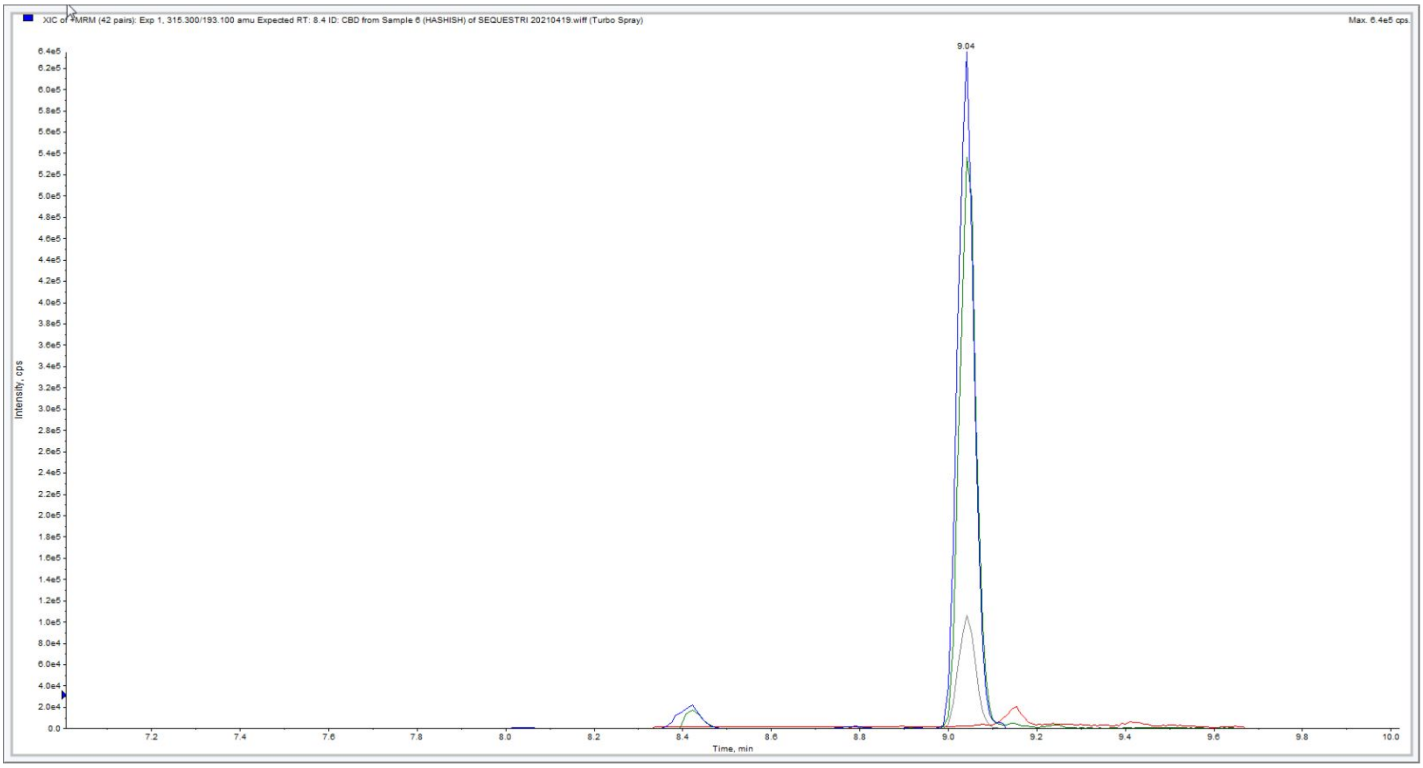

Identified material: MARIJUANA

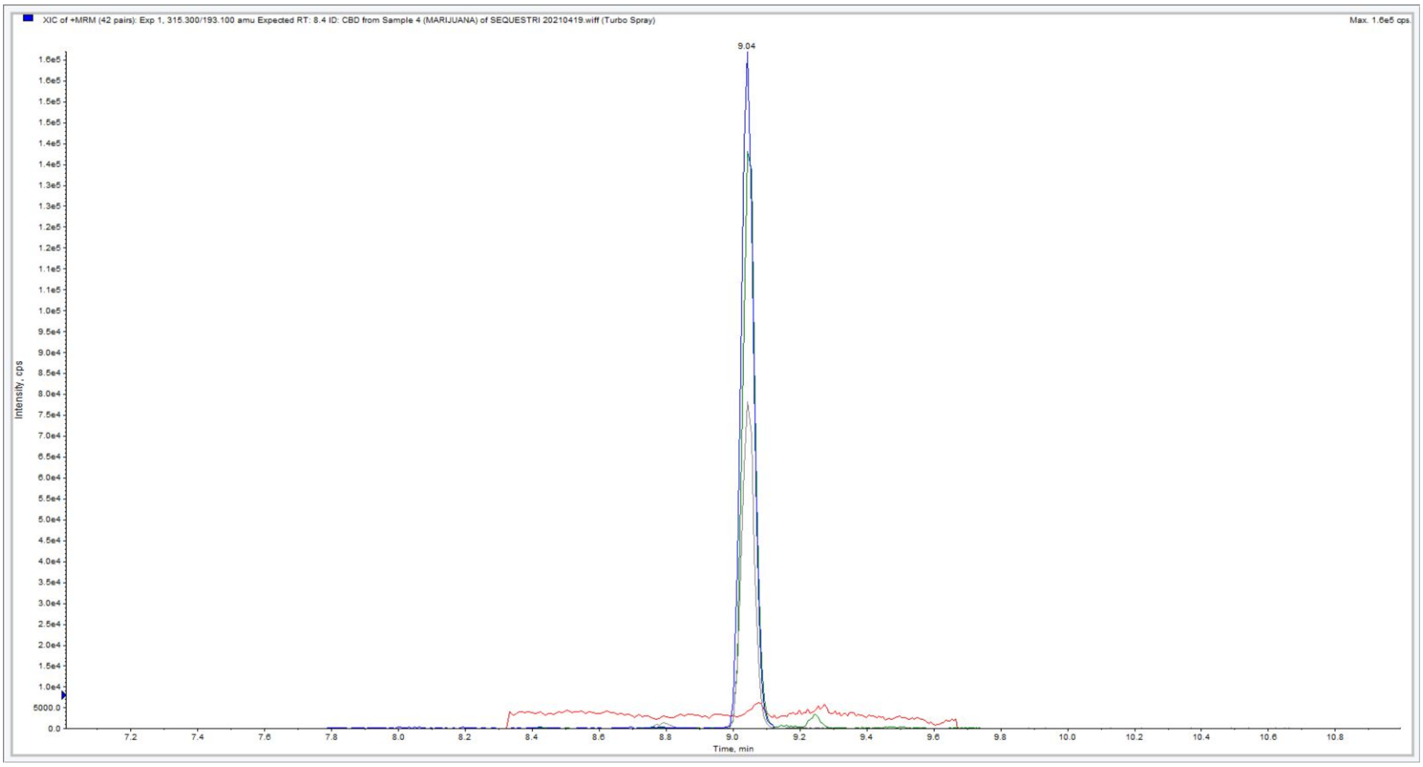

Identified material: HEROIN

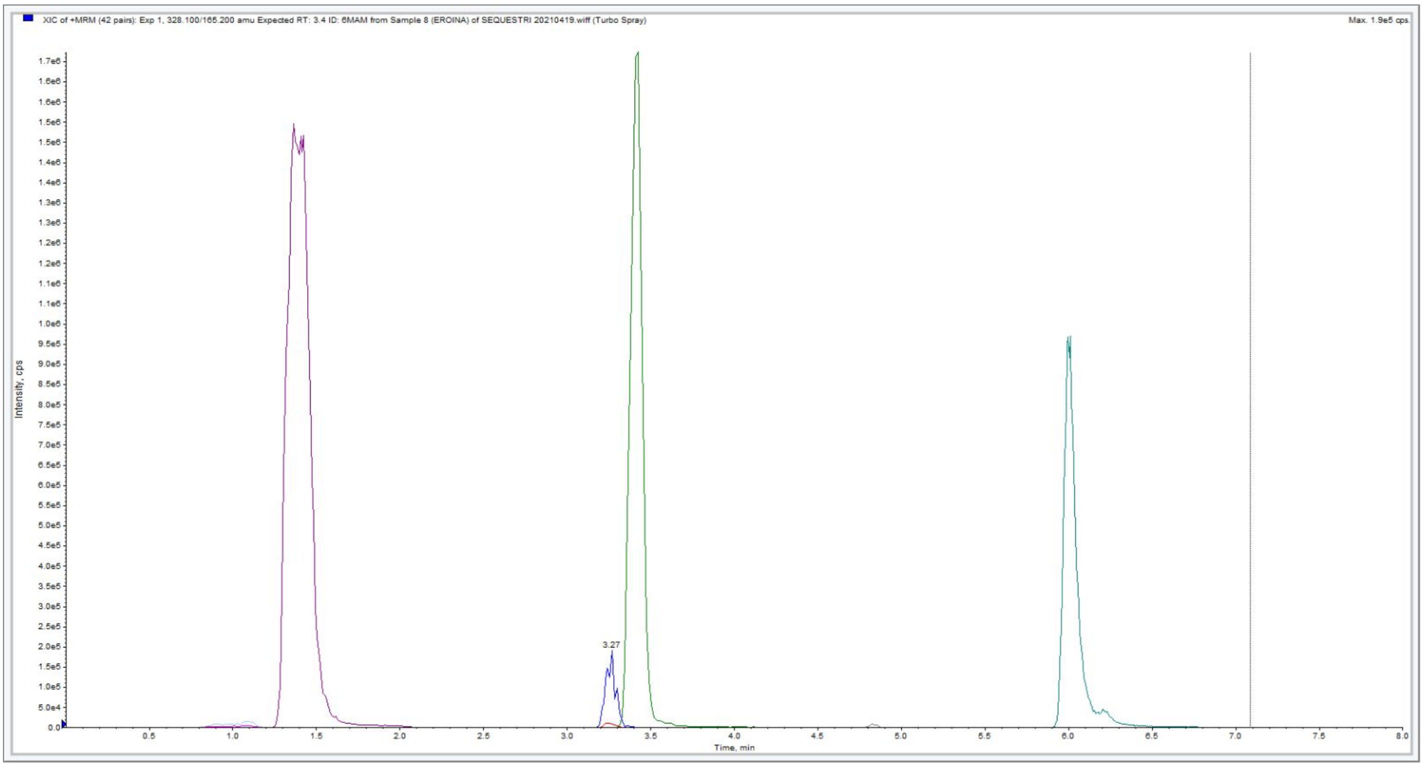

Identified material: 3,4-METHYLENEDIOXYMETHAMPHETAMINE or MDMA

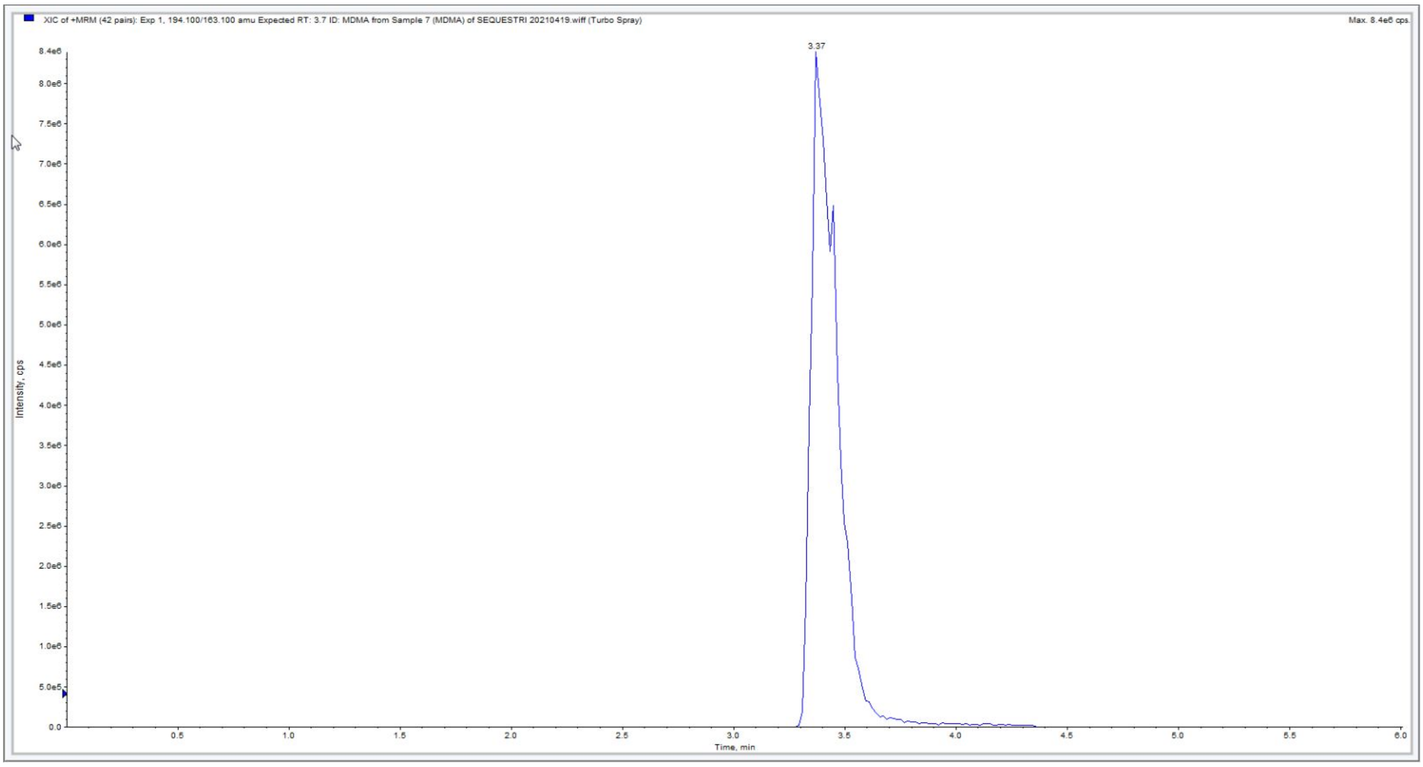

Table S.6 Comparison with other methods in literature

Analytes	Matrix	Treatment	Instrumentation	Runtime	LOD/LOQ	Ref.
Methamphetamine	Seized street samples	-	Surface-enhanced Raman sensing	-	LOD 7 pM	(1)
Cocaine, heroin, THC	Solid seized material	Dilution	GC-MS (qualitative)<br>GC-FID (quantitative)	20 min<br>12 min	LOD 3 ng/µL<br>LLOQ 4.2 ng/µL	(2)
Cl^{-}, NO_2^{-}, NO_3^{-}, SO_4^{2-}, CO_3^{2-}, BO_3^{3-}, Levamisole, Lidocaine, Cocaine	Solid seized material	Dilution	CE-UV	3 min	LOD 1.5-3 x 10^{-2} mmol/L<br>LOQ 0.05-1.2 mmol/L	(3)
Cathinones, synthetic cannabinoids, miscellanea (32 compounds)	Solid seized material	Dilution	GC-MS<br>LC-HRMS	17.8 min<br>18.5 min	Qualitative analyses, no LOD and LOQ	(4)
Alprazolam, amphetamine, aminopyrine, benzocaine, caffeine, cocaine, codeine, diltiazem, ephedrine, fentanyl, fenethylline, furanylfentanyl, heroin, hydroxyzine, levamisole, lidocaine, methamphetamine, morphine, noramidopyrine, phencyclidine, phenacetin, procaine, strychnine, xylazine	Solid seized material	Dilution	Portable GC-MS	15 min	Qualitative analyses, LOD from 0.01 to 0.1 mg/mL, no LOQ	(5)
Amphetamine	Solid seized material	Dilution derivatization	Portable SWV	-	LOD 22.2 µM<br>LOQ 50 µM	(6)
54 different fentanyl analogues	Standard solution	-	GC-MS	16 min	PCA cluster classification	(7)
Noscapine, heroin, papaverine, acetyl thebaol	Seized heroin samples	-	LC-MS/MS	6 min	LOD 0.4 mg/L<br>LOQ 1.0 mg/L	(8)

Table S.6 cont. Comparison with other methods in literature

Analytes	Matrix	Treatment	Instrumentation	Time	LOD/LOQ	Ref.
Fentanyl	Seized drug samples	-	LFIA	-	Qualitative analyses	(9)
Ketamine	Simulated street samples	Dilution	Portable potentiostat (voltammetry)	-	Qualitative analyses	(10)
Methamphetamine, ketamine, cocaine, and cutting agents	Seized drug samples	Solid analysis	Raman spectroscopy	-	Qualitative analyses, LOD from 5% to 20% of the material	(11)
MDMA	Seized ecstasy tablets	Dilution	LC-DAD	15 min	LOD 0.7 μg/mL<br>LOQ 3.1 μg/mL	(12)
Cocaine, buprenorphine, amphetamine, methamphetamine, MDMA, MDA, MDE, MBDB, ketamine, diacetylmorphine, ephedrine, pseudoephedrine, methadone, methorphan, 6-MAM, THC, morphine, acetylsalicylic acid, aminophenazone, benzocaine, caffeine, diltiazem, phenacetin, ibuprofen, hydroxyzine, levamisole, lidocaine, naloxone, nicotine, noscapine, paracetamol, paroxetine, procaine, procainamide, trimetoprim, sulfametoxazole, tropacocaine	Seized solid and liquid samples	Dilution	LC-MS/MS	15 min	LOD 1.67 ng/mL<br>LOQ 5 ng/mL	Current validated method

PP: protein precipitation; LC-MS/MS: liquid chromatography-tandem mass spectrometry; GC-MS: gas chromatography-mass spectrometry; GC-FID: gas chromatography-flame ionization detector; CE-UV: capillary electrophoresis-spectrophotometric detector; LC-HRMS: liquid chromatography-high resolution mass spectrometry; SWV: square wave voltammetry sensor; LFIA: later flow immunoassay;

References

(1) Mao, J.; Kang, Y.; Yu, D.; Zhou, J. *Anal. Chim. Acta* **2021**, 1146, 124-130.

(2) Silvestre, A.; Basilicata, P.; Coraggio, L.; Guadagni, R.; Si-monelli, A.; Pieri, M. *Forensic Sci. Int.* **2021**, 321, 110738.

(3) Barreto, D.N.; Ribeiro, M.M.A.C.; Sudo, J.T.C.; Richter, E.M.; Muñoz, R.A.A.; Silva, S.G. *Talanta* **2020**, 217, 120987.
(4) Odoardi, S.; Romolo, F.S.; Strano-Rossi, S. *Forensic Sci. Int.* **2016**, 265, 116-120.
(5) Fiorentin, T.R.; Logan, B.K.;, Martin, D.M.; Browne, T.; Rieders, E.F. *Forensic Sci. Int.* **2020**, 313, 110342.
(6) Parrilla, M.; Montiel, N.F.; Van Durme, F.; De Wael, K. *Sens. Actuators B Chem.* **2021**, 337, 129819
(7) Gilbert, N.; Mewis, R.E.; Sutcliffe, O.B. *Forensic Chem.* **2020**, 21, 100287.
(8) Jovanov, P.; Petrin-Miličević, M.; Radosavljević-Stevanović, N.; Vraneš, M.; Belić, S.; Sakač, M.; Nikolov, J.; Gadžurić, S. *Anal. Lett.* **2021**, 54(7), 1224-1232.
(9) Angelini, D.J.; Biggs, T.D.; Prugh, A.M.; Smith, J.A.; Han-burger, J.A.; Llano, B.; Avelar, R.; Ellis, A.; Lusk, B.; Naanaa, A.M.; Sisco, E.; Sekowski, J.W. *J. Forensic Sci.* **2021**, 66, 758–765.
(10) Schram, J.; Parrilla, M.; Sleegers, N.; Samyn, N.; Bijvoets, S.M.; Heerschop, M.W.J. *Anal. Chem.* **2020**, 92, 13485−13492.
(11) Liu, C.-M.; He, H.-Y.; Xu, L.; Hua, Z.-D. *Drug Test Anal.* **2021**, 13, 720–728.
(12) Duarte, L.O.; Ferreira, B.; Silva, G.R.; Ipólito, A.J.; de Oli-veira, M.F. *J. Liq. Chromatogr. Relat. Technol.* **2021**, in press.

Table S.7 General classification for GAPI index

	Category			Note
	Green	Yellow	Red	
**Sample preparation**				
Collection (1)				*Off-line*
Preservation (2)				None
Transport (3)				Required
Storage (4)				None
Type of method: direct or indirect (5)				Simple treatments
Scale of extraction (6)				Microextraction
Solvents/reagents used (7)				Green solvents/reagents used
Additional treatments (8)				None
**Reagents and Solvents**				
Amount (9)				<10 mL (< 10 g)
Health hazard (10)				Slightly toxic, slight irritant; NFPA health hazard score = 0 or 1.
Safety hazard (11)				Highest NFPA flammability or instability score of 0 or 1. No special hazards.
**Instrumentation**				
Energy (12)				≤1.5 kWh per sample
Occupational hazard (13)				Hermetic sealing of analytical process
Waste (14)				1-10 mL (1-10 g)
Waste treatment (15)				No treatment
**Additional Mark: Quantification**				
Circle in the middle of GAPI: procedure for quantitative and qualitative analysis				

Figure S.4 Correspondence to GAPI pictogram in the main text:

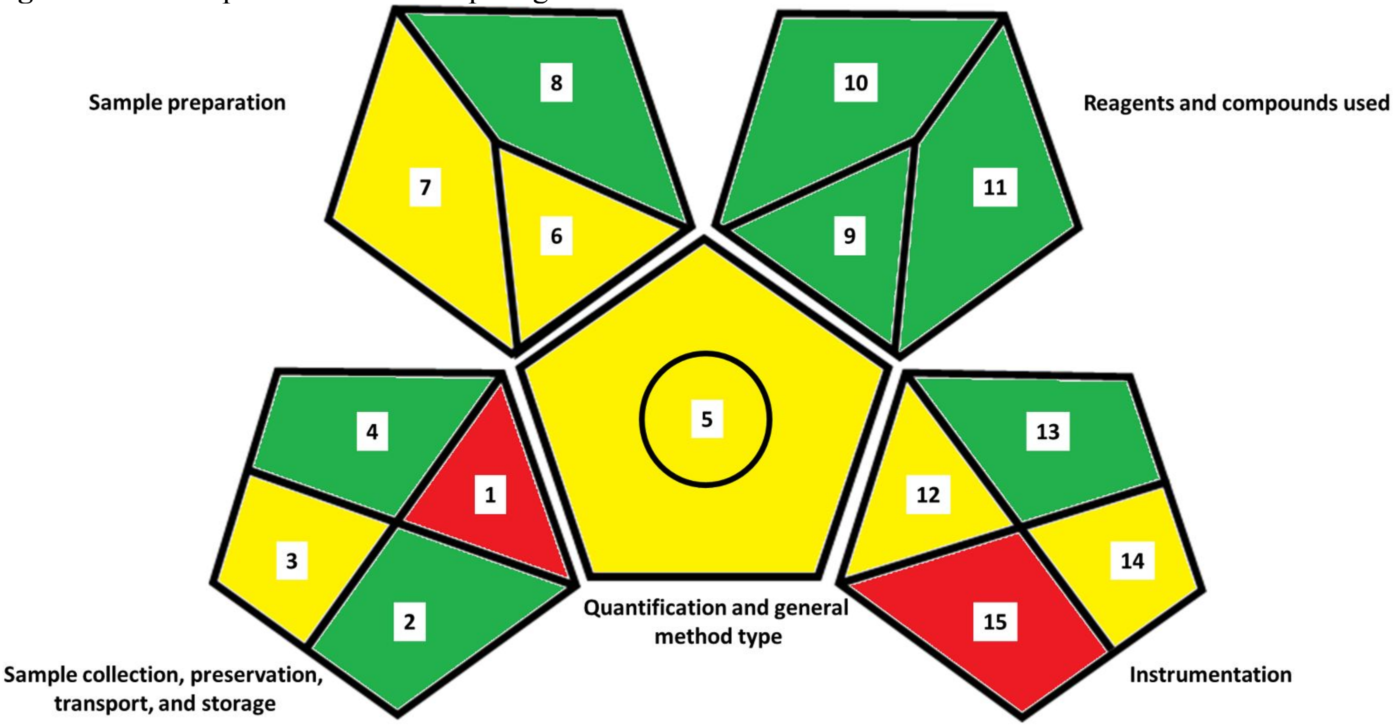

Supplement: Supplementary file 1 — ac1c03310_si_001.pdf [file ac1c03310_si_001.pdf]
